# Enhancing NMR derived ensembles with kinetics on multiple timescales

**DOI:** 10.1007/s10858-019-00288-8

**Published:** 2019-12-14

**Authors:** Colin A. Smith, Adam Mazur, Ashok K. Rout, Stefan Becker, Donghan Lee, Bert L. de Groot, Christian Griesinger

**Affiliations:** 1 Department for Theoretical and Computational Biophysics, Max Planck Institute for Biophysical Chemistry, Göttingen, Germany; 2 Department for NMR-Based Structural Biology, Max Planck Institute for Biophysical Chemistry, Göttingen, Germany; 3 Department of Chemistry, Wesleyan University, Middletown, USA; 4 James Graham Brown Cancer Center, University of Louisville, Louisville, USA; 5 Present Address: Biozentrum, University of Basel, Basel, Switzerland

**Keywords:** Nuclear overhauser effect, Protein dynamics, Ensemble, Structure determination, Kinetics

## Abstract

Nuclear magnetic resonance (NMR) has the unique advantage of elucidating the structure and dynamics of biomolecules in solution at physiological temperatures, where they are in constant movement on timescales from picoseconds to milliseconds. Such motions have been shown to be critical for enzyme catalysis, allosteric regulation, and molecular recognition. With NMR being particularly sensitive to these timescales, detailed information about the kinetics can be acquired. However, nearly all methods of NMR-based biomolecular structure determination neglect kinetics, which introduces a large approximation to the underlying physics, limiting both structural resolution and the ability to accurately determine molecular flexibility. Here we present the Kinetic Ensemble approach that uses a hierarchy of interconversion rates between a set of ensemble members to rigorously calculate Nuclear Overhauser Effect (NOE) intensities. It can be used to simultaneously refine both temporal and structural coordinates. By generalizing ideas from the extended model free approach, the method can analyze the amplitudes and kinetics of motions anywhere along the backbone or side chains. Furthermore, analysis of a large set of crystal structures suggests that NOE data contains a surprising amount of high-resolution information that is better modeled using our approach. The Kinetic Ensemble approach provides the means to unify numerous types of experiments under a single quantitative framework and more fully characterize and exploit kinetically distinct protein states. While we apply the approach here to the protein ubiquitin and cross validate it with previously derived datasets, the approach can be applied to any protein for which NOE data is available.

**Electronic supplementary material:**

The online version of this article (10.1007/s10858-019-00288-8) contains supplementary material, which is available to authorized users.

## Introduction

The development of methods for determining the structure and dynamics of proteins has been instrumental in elucidating mechanisms behind many important functions, including ligand binding, enzyme-catalysis, allosteric regulation, and folding. Through the use of several nuclear magnetic resonance (NMR) effects, including scalar couplings, longitudinal/transverse relaxation (T_1_/T_2_), the heteronuclear nuclear Overhauser effect (NOE), residual dipolar couplings, cross-correlated relaxation, and relaxation dispersion, an extraordinary amount of atomic resolution information can be extracted about the local structure and dynamics of proteins. However, to build a more complete picture of the thermodynamically accessible states of a protein, the determination of an ensemble of atomic resolution structures is often essential. In that regard, homonuclear NOEs, which give information about through space interactions between protons, are of critical importance and have been used in nearly all high-resolution structures determined by NMR.

Most NMR structures are solved using distance-based methods typically considered to be semiquantitative (Borgias and James 1988; LeMaster et al. 1988), in which NOE cross peaks are analyzed with the isolated spin pair approximation (ISPA) that assumes any transfer of magnetization between protons happens solely through space and does not involve the network of neighboring protons. Cross-peaks are often categorized as strong, medium, and weak, then used to derive approximate upper bound distance restraints from which individual structures are solved through computational refinement. Most NMR ensembles deposited in the Protein Data Bank (PDB) consist of about 20 of the best individually determined structures. Although the heterogeneity in these ensembles may reflect true protein flexibility, it often results from the uncertainty due to lack of sufficient restraints or limitations of the modeling procedures.

Several techniques have been employed to enable the determination of more accurate structures and ensembles from NMR data. In the decade around which the first NMR structure was published (Williamson et al. 1985), there was much interest in applying techniques that did not employ the ISPA, but instead treated the full matrix of possible magnetization transfer pathways through neighboring protons (Borgias and James 1988; Keepers and James 1984; Boelens et al. 1988; Yip and Case 1989; Post et al. 1990; Bonvin et al. 1991; Bonvin et al. 1993). However, in most of these approaches, a single conformation was assumed to be a faithful representative of the structural ensemble of the protein. More recently, relaxation matrix calculations were used to reweight an ensemble of conformations using maximum entropy methods (Vasile 2019). Furthermore, other efforts have been made to incorporate additional experimental information into the determination of NMR ensembles, either using RDC (Cornilescu et al. 1998; Lange et al. 2008; Frank et al. 2009; Fenwick et al. 2011; Montalvao et al. 2012; Maltsev et al. 2014) or fast timescale order parameter (S^2^) (Lindorff-Larsen et al. 2005; Richter et al. 2007; Chen et al. 2007) data. These efforts incorporated one or more of the following features which were probably important to their success: addition of experimental data that directly captures protein dynamics, fitting experimental data to multiple structures simultaneously, and the use of explicit solvent molecular dynamics (MD) refinement with high quality force fields. More recently, the exact NOE (eNOE) approach (Vögeli et al. 2009; Vögeli et al. 2010) has used diagonal peaks, buildup curves, and perdeuteration or relaxation matrix analysis to more accurately determine NOE rates. This has allowed much more precise interatomic distances to be inferred and enabled the determination of multi-state protein ensembles (Vögeli et al. 2012; Chi et al. 2015; Vögeli et al. 2016). However, a notable drawback of nearly all NOE structure determination efforts to date, including the eNOE approach, is that a distinction of time scales for NOE averaging was not attempted and that angular motion is neglected during refinement and at best can only be derived in a post hoc manner using an independently determined reference structure (Vögeli et al. 2009).

This omission is notable because it has been shown that determination of effective internuclear distances requires taking into account fluctuations in the internuclear orientation, and that this can be affected by anisotropic internal motions typical in proteins (LeMaster et al. 1988). One means of incorporating both radial and angular internal dynamics is to explicitly calculate internuclear correlation functions and the resulting NOE rates from molecular dynamics trajectories (Bonvin et al. 1993; Brueschweiler et al. 1992; Peter et al. 2001; Chalmers et al. 2016). While this approach can be used for simulating NMR data and validating MD simulations, it has limited applications in ensemble determination. One notable hybrid approach involved using internuclear order parameters estimated from MD simulations to scale NOE rates calculated from an ensemble of structures (Bonvin et al. 1993). However, this method depends on having an accurate MD simulation and assumes distances average as r^−6^. Furthermore, it does not guarantee that the resulting ensemble will have accurate angular dynamics, or even the same angular dynamics as the initial MD simulation.

The importance of angular dynamics in proteins has long been appreciated. Through the use of model free (Lipari and Szabo 1982) and extended model free approaches (Clore et al. 1990), along with associated experimental advances (Kay et al. 1989), the determination of Lipari-Szabo order parameters (S^2^) has become the most widely applied method for elucidating protein dynamics. These values give the amplitude of angular fluctuations of bond vectors within the protein. The strong dependence of the heteronuclear NOE between atoms (e.g. the backbone amide nitrogen/hydrogen) on the amplitude of their relative angular motion, together with T_1_/T_2_ measurements, is critical for determining order parameters. The larger the amplitude, or the shorter the timescale of that motion, the more the transfer of magnetization via the NOE is attenuated. This same phenomenon is at work in homonuclear NOE experiments used to determine interatomic distances.

The approach described here, which we call Kinetic Ensemble (KE), explicitly models both the amplitudes and timescales of angular motion involved in homonuclear NOEs. It involves describing atomic motions using a lattice defined by the structural ensemble. Previous studies have used the lattice approach to model toy systems with specialized or approximate spectral density functions (Keepers and James 1984; Brueschweiler et al. 1992; Koning et al. 1990; Liu et al. 1992), making notable contributions to the understanding of methyl rotation and aromatic ring flips (Koning et al. 1990; Liu et al. 1992). The theory described here is a generalization of both the lattice and model free approaches that can handle complex combinations of angular and radial motions in a rigorous manner. The importance of hierarchical protein dynamics has been previously noted (Henzler-Wildman et al. 2007; Smith et al. 2015; Lewandowski et al. 2015), and several techniques have been recently developed to reweight multi-timescale models built from extensive molecular dynamics simulations (Salvi et al. 2016; Wan et al. 2016; Capelli et al. 2018). By contrast, our approach is better suited for modeling such temporally resolved dynamics with an ensemble representation typically used to represent NMR structures, as demonstrated on a protein system using up to five different timescales of molecular motion. We show that the timescale of distance fluctuations has a large impact on how those distances are averaged over a much broader range of timescales than previously appreciated. This is shown to be critical for interpreting NOE data in the context of several state-of-the-art protein ensembles. Despite this, our approach is general and can be applied to ensembles derived solely from NOEs acquired at a single mixing time and does not explicitly require other NMR data.

## Results

### Kinetic ensemble (KE) approach can be simplified to extended model free formalism

**Fig. 1:**
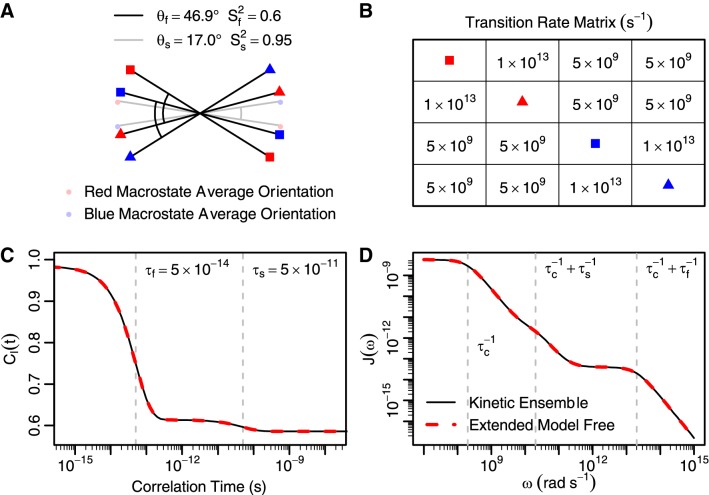
Kinetic Ensemble method can recapitulate Extended Model Free approach **a** A fixed-distance double atom system showing motion on two timescales with different magnitudes of motion (fast: S_f_^2^, slow: S_s_^2^) can be represented by four microstates with an angle (θ_f_) between the two pairs of quickly interconverting microstates and another angle (θ_s_) between the average orientations of the two slowly exchanging macrostates. Overall molecular tumbling is ignored here. **b** In such a system, the slow timescale (τ_s_) can be represented with inter-macrostate rates equal to τ_s_^−1^/4. The fast timescale (τ_f_) is captured by an intra-macrostate rate of (τ_f_^−1^−1/2τ_s_^−1^)/2. **c** When the internal correlation function for this Kinetic Ensemble is calculated, it is identical to Extended Model Free. **d** The same is true for the resulting spectral density function, from which NOE rates are determined

To validate the Kinetic Ensemble approach using a multi-timescale scheme, we used a two-atom, fixed bond length system with two slowly interconverting macrostates, each consisting of two quickly interconverting microstates (Fig. 1). If constructed correctly, this system should recapitulate extended model free formalism (see “Methods”) (Clore et al. 1990). The conformations of the four states were constructed by rotating the two-atom system within a plane. For each macrostate, the angle (θ_f_) between the two microstates was set such that the fast timescale order parameter (S_f_^2^) was 0.6. The angle (θ_s_) between the average orientations of the macrostates was set such that the overall order parameter of the system (S^2^) was 0.57, giving a slow timescale order parameter (S_s_^2^ = S^2^/S_f_^2^) of 0.95. A transition rate matrix (Q) was constructed to recapitulate fast and slow timescales of motion (τ_f_ and τ_s_, respectively, Fig. 1b). The diagonal elements were set to the negative sum of the other elements in the corresponding row (not shown). An eigendecomposition of the resulting matrix yields three unique eigenvalues (0, degenerate − τ_f_^−1^, and − τ_s_^−1^), indicating two distinct timescales of motion. Upon application of the Kinetic Ensemble approach, the resulting internal correlation functions and spectral densities exactly match those of extended model free theory (Clore et al. 1990), with $\tau_{c} = 5 \times 10^{ - 9} {\text{s}},\;\tau_{f}^{\prime } = \tau_{f} \tau_{c} /\left( {\tau_{f} + \tau_{c} } \right)\;{\text{and}}\;\tau_{s}^{\prime } = \tau_{s} \tau_{c} /\left( {\tau_{s} + \tau_{c} } \right):$

$$C_{I} \left( t \right) = S_{f}^{2} S_{s}^{2} + \left( {1 - S_{f}^{2} } \right)e^{{ - t/\tau_{f} }} + S_{f}^{2} \left( {1 - S_{s}^{2} } \right)e^{{ - t/\tau_{s} }}$$

$$J\left( \omega \right) = 2\left( {\frac{{S_{f}^{2} S_{s}^{2} \tau_{c} }}{{1 + \omega^{2} \tau_{c}^{2} }} + \frac{{\left( {1 - S_{f}^{2} } \right)\tau_{f}^{\prime } }}{{1 + \omega^{2} \tau_{f}^{\prime 2} }} + \frac{{S_{f} \left( {1 - S_{s}^{2} } \right)\tau_{s}^{\prime } }}{{1 + \omega^{2} \tau_{s}^{\prime 2} }}} \right)$$

As this analysis shows, the Kinetic Ensemble approach simplifies to extended model free when the rate matrix has only three distinct eigenvalues and bond lengths are kept fixed. However, it also handles arbitrarily complex kinetic schemes and bond length variability, as discussed below.

### Dependence of distance averaging on exchange timescale

**Fig. 2:**
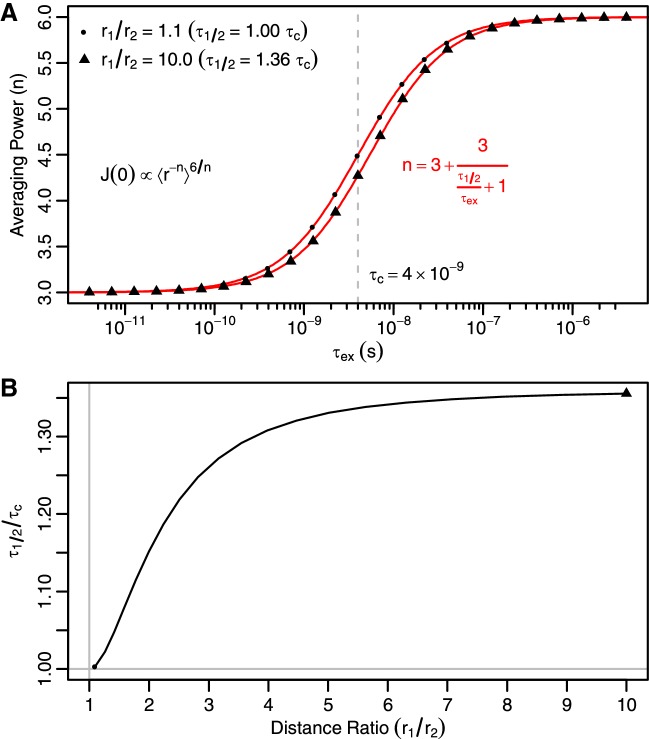
Interatomic distance averaging transitions smoothly from a power of three to six **a** In a double-atom system with states at two distinct distances (r_1_ and r_2_), the Kinetic Ensemble approach can be used to understand the dependence of the spectral density function, J(0), on how the averaging of interatomic distances (r) is influenced by the relative timescales of exchange (τ_ex_) and overall tumbling (τ_c_). The black points were determined by fitting the black equation to simulations with different populations of the two states. Simulations with τ_ex_ ≪ τ_c_ give the expected ⟨r^−3^⟩^2^ dependence and τ_ex_ ≫ τ_c_ give the expected ⟨r^−6^⟩ dependence. Between those two limits, there is a smooth transition that empirically follows a hyperbolic equation (red). Depending on the ratio of the two distances (r_B_/r_A_), the hyperbolic function shifts right or left between the two extremes shown, with τ_1/2_ being the timescale with an averaging power of 4.5. **b** As the distance ratio increases, τ_1/2_ goes from 1.00τ_c_ to a plateau value of 1.36τ_c_

As is clear from extended model free theory, the particular timescales at which molecules undergo angular reorientation has a large influence on the spectral densities and resulting experimental observables. The same is true for the timescales at which distances change. For motions occurring much faster than molecular tumbling, the NOE rate is known to be dependent on the r^−3^ average of interatomic distances (r), while motions occurring much slower show r^−6^ distance averaging. Mathematically, that can be expressed $J\left( 0 \right) \propto \left\langle {r^{ - 3} } \right\rangle^{2}$ for fast motion and $J\left( 0 \right) \propto r^{ - 6}$ for slow motion, assuming the NOE rate is dominated by the $J\left( 0 \right)$ term, which is true for both the auto (ρ) and cross (σ) relaxation rates of macromolecules. The Kinetic Ensemble approach should implicitly capture this dependence. To test this, we constructed a model system having two states with the same orientation but different distances: one (r_A_) fixed at 1 Å and the other (r_B_) varied 1.1–10 Å. While τ_c_ was kept fixed, the exchange timescale (τ_ex_) was varied around τ_c_ plus or minus three orders of magnitude. For each value of τ_ex_, the Kinetic Ensemble approach was used to calculate the spectral density function with the population of the first state (*p*_A_) set to 0, 0.2, 0.4, 0.6, 0.8, and 1.0. At each τ_ex_ value, we used the equation $J\left( 0 \right) = 2\tau_{c} \left( {p_{\text{A}} r_{\text{A}}^{ - n} + \left( {1 - p_{A} } \right)r_{\text{B}}^{ - n} } \right)^{6/n}$ to fit the effective averaging power (*n*). The resulting values of *n* are shown in Fig. 2a. When τ_ex_ is at least two orders of magnitude away from τ_c_, the usual assumptions of distancing averaging are valid. However, at intermediate timescales the averaging power smoothly transitions from three to six. The surprisingly shallow slope of this function at τ_c_ indicates that this distance dependence could be used to extract information about dynamics at least an order of magnitude slower than molecular tumbling. This finding agrees with previous work indicating NOE sensitivity to dynamics plus or minus an order of magnitude around τ_c_ (Brueschweiler et al. 1992). We found that the averaging powers could be empirically fit with a hyperbolic function similar to that used for bimolecular binding, with the midpoint τ_1/2_:

$$n = 3 + 3/\left( {\frac{{\tau_{ 1 / 2} }}{{\tau_{\text{ex}} }} + 1} \right)$$

The previous two examples illustrate a clear timescale dependence for dipole–dipole interactions between nuclei undergoing either angular reorientation or changes in distance. In a macromolecule, both types of motion are prevalent and convoluted together, precluding the use of simple analytical models (Fig. S4). Whereas most macromolecular modelling efforts neglect one or both types of motion, assume that they are separable, or make implicit assumptions about the timescale, the Kinetic Ensemble approach models the effects of motion in a rigorous manner subject to the structural ensemble and transition rate matrix.

### Much internal protein motion occurs at a timescale similar to overall tumbling

To apply the Kinetic Ensemble approach to a more complex biomolecular system, we used the protein ubiquitin. Using high-resolution 2D NOESY experiments, we acquired a set of buildup curves with 34 equally spaced mixing times from 5 to 500 ms at a temperature of 308 K. The signal to noise of this dataset was excellent and showed a high degree of reproducibility in duplicate experiments. To ensure that we had a good basis set of physically realistic ubiquitin structures, we initially analyzed the EROS3 ensemble (Lange et al. 2008), which was generated by refining randomly selected 8 member subsets of 46 crystal structures against NOE and RDC data using explicitly solvated molecular dynamics simulations, with the best 22 subsets selected to produce 176 total ensemble members.

For modeling ubiquitin, we started using a transition rate matrix with the fewest parameters possible, assuming a single timescale of motion with a uniform transition rate between all ensemble members (i.e. an N-site jump model). This poses the least risk for overfitting, while giving the flexibility for the model to explore averaging regimes between r^−3^ and r^−6^, and varying amounts of NOE attenuation due to angular dynamics. Given an overall exchange timescale, τ_ensemble_, the individual transition rate, k, can be calculated k = τ_ensemble_^−1^/N, with number of ensemble members, N = 176. It is trivial to verify the overall exchange timescale, as the eigenvalues of the transition rate matrix contain a single value of 0 and 175 degenerate values equal to − τ_ensemble_^−1^. In addition to interconversion between ensemble members, methyl and aromatic group rotations were accounted for as independent exchange processes (see “Methods”).

**Fig. 3:**
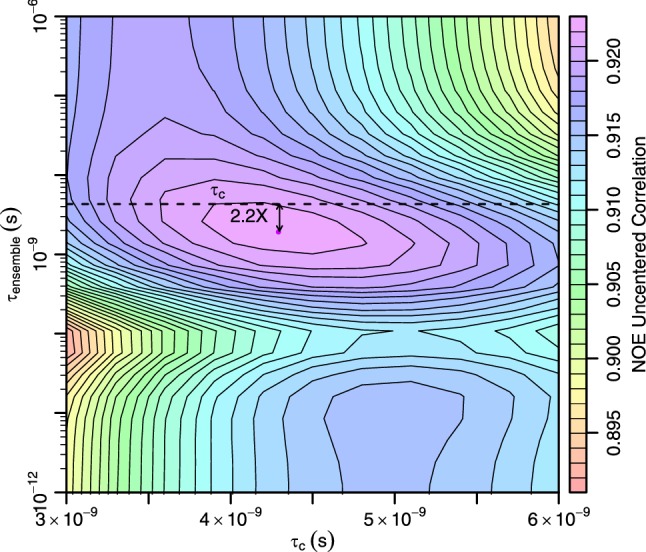
Optimization of time coordinates gives similar tumbling and exchange timescales. Kinetic Ensemble Refinement optimizes the timescale of molecular tumbling (τ_c_) and one or more exchange timescales (in this case just τ_ensemble_). When the two are simultaneously optimized for the EROS3 ensemble (purple point), the τ_c_ is 4.3 ns, close to previously published tumbling times for ubiquitin. The best τ_ensemble_ is 2.0 ns, which is within 2.2 times faster than τ_c_ (indicated as a dashed line on the τ_ensemble_ axis). According to Fig. 2, having τ_ensemble_ within 2.2-fold of τ_c_ makes the effective averaging of distances a power of 3.8–4.9, contrary to usual approximations of NOE data that assume powers of either three or six

To determine the timescales that best fit the experimental data, we sampled τ_c_ and τ_ensemble_ on a grid and computed the uncentered correlation with experimental NOE data at each point (Fig. 3). The uncentered correlation is very similar to the Pearson correlation but penalizes data with a non-zero y-intercept (see “Methods”). The agreement with experimental data was much more sensitive to τ_c_, with large decreases in correlation visible with a less than twofold deviation in τ_c_ from the optimal value. While τ_ensemble_ showed comparatively less sensitivity over a much larger range (± 3 orders of magnitude), a distinct peak was seen at around 2.0 ns. After sampling on the grid, the timescales from the best fitting point were numerically optimized to give the best fitting τ_c_ value of 4.3 ns. Previous measurements of ubiquitin at 308 K have given very similar tumbling times of 4.0 ns (Sabo et al. 2012), suggesting the protocol can accurately determine kinetics from NOE data. Similarly, an overall ensemble timescale of 2.0 ns is supported by a comparison of backbone amide Lipari-Szabo order parameters and RDC-based order parameters. Previous studies (Lange et al. 2008; Lakomek et al. 2008) have shown that the majority of angular fluctuations throughout ubiquitin are captured by Lipari-Szabo order parameters ($S_{LS}^{2}$), which report on motions close to or faster than τ_c_, but some residues show a substantial amount of additional motion ($S_{rdc}^{2}$/$S_{LS}^{2}$) in RDC-based order parameters ($S_{rdc}^{2}$), which report on dynamics up to the millisecond timescale. 2.0 ns would therefore be a reasonable compromise fit of a single exchange timescale to both the sub and supra-τ_c_ motions. Motions with timescales much slower than τ_c_ affect the NOE by upweighting the contributions of short contacts (through r^−6^ averaging) without having additional angular attenuation beyond that occurring at fast timescales.

### Modeling an extended hierarchy of timescales

**Fig. 4:**
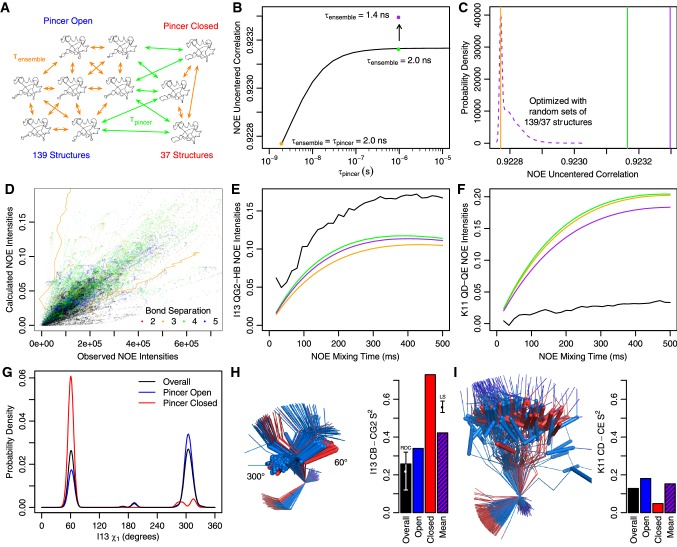
Hierarchical kinetics improve fit to NOE data. **a** In the hierarchical scheme, different transition rates are used between and within groups of ensemble members (here pincer open and closed). **b** Starting from a single timescale (orange) slowing down the ubiquitin pincer mode (τ_pincer_) improves correlation with NOE data (green). Subsequent optimization of the ensemble timescale (τ_ensemble_) further improves the correlation (purple). **c** Applying the same optimization protocol to 20,000 sets of randomized groups shows that the enhancement from grouping by pincer state is very significant. **d** Experimental NOE intensities and theoretical NOE buildups fit to a single timescale. Nearly all NOEs highly overestimated by the model come from those between protons separated by 2–5 bonds (bond separations ≥ 6 are black). Buildups illustrated in **e** and **f** are drawn with orange lines. Calculated NOEs show the summed fractional magnetization transfer and observed are arbitrary units, with the limits of the graph set to three times the root mean square (RMS) value. **e** The I13 QG2-HB buildup showed the greatest improvement after optimization of only τ_pincer_ (green), compared with the RMS-normalized experiment (black). It slightly worsens after optimization of τ_ensemble_ (purple). **f** The K11 QD-QE buildup showed the greatest improvement after τ_ensemble_ optimization. **g** The I13 shows more restricted sampling of chi angles when just open or closed conformations are considered. H) This is mirrored when looking at the I13 structures or CB-CG2 order parameters (S^2^). The side chains have their CA atoms aligned and the CB-CG2 bond shown with a stick representation. When τ_pincer_ is much greater than τ_c_, the population weighted mean order parameter dominates in determining the NOE rate, which results in a net increase in NOE intensities after both timescales are optimized. The overall and mean values compare well to previously determined RDC (Fares et al. 2009) and Lipari-Szabo (Lee et al. 1999) order parameters. **i** The K11 CD-CE bond vector is more dynamic and shows a much smaller increase in mean order parameter, resulting in a net decrease in calculated NOE intensities

After already modelling a single ensemble timescale, we sought to test whether separating out another, slower ensemble motion would further improve the model. Much of the supra-τ_c_ motion in ubiquitin has been attributed to the pincer mode (Lange et al. 2008), which involves opening and closing around the β1–β2 loop and the end of the alpha helix. Extensive relaxation dispersion data strongly suggests that this motion is faster than 3.4 µs (Massi et al. 2005; Smith et al. 2016), giving a range of approximately three orders of magnitude for the kinetics of this motion. Furthermore, even if that level of characterization had not been done, the pincer mode is the largest amplitude motion observed in unbiased MD simulations (Peters and de Groot 2012), suggesting questions about its timescale. This provides a good test case to determine whether NOE data can be better modeled using multiple timescales of internal motions. To compose such a hierarchy as illustrated in Fig. 4a, the overall transition rate matrix was constructed with inter-group rates set to τ_pincer_^−1^/N, with N being the total number of structures. The intra-group rates were set to (τ_ensemble_^−1 ^− (1−N_i_/N)τ_pincer_^−1^)/N_i_, with N_i_ being the number of structures in the given group (in this case 139 open and 37 closed).

Eigendecompositon of this rate matrix yields exactly three distinct eigenvalues, 0, − τ_pincer_^−1^, and − τ_ensemble_^−1^. The rate matrix in Fig. S2a was constructed in the same manner. Keeping τ_ensemble_ fixed at 2 ns, the optimal correlation was found by sampling τ_pincer_ on an interval from 2 ns to 1 µs. (Fig. 4b) The correlation improved as the timescale slowed, plateauing at about 100 ns and reaching a maximum at the limit of 1 µs. While a 1 µs pincer motion might be partially visible in relaxation dispersion curves having 3.4 µs resolution, this was not observed experimentally. Nevertheless, because NOE rates are largely insensitive to timescale differences in the regime 25–250 times slower than overall tumbling (i.e. 100 ns–1 µs here), there is little practical effect in using a τ_pincer_ somewhat slower than experimentally justified. Using a τ_pincer_ of 1 µs, τ_ensemble_ was then numerically reoptimized from 2 ns to 1.4 ns. This decrease in timescale was likely enabled by reducing the amount of slow timescale “compromise” required. To determine whether the overall increase in uncentered correlation was statistically significant, we repeated that same optimization procedure 20,000 times using randomly grouped sets of 139 and 37 structures. (Fig. 4c) None of these random sets showed improvements greater than the pincer grouping, suggesting a p ≪ 5 × 10^−5^.

Most previous efforts to model homonuclear NOE data have focused solely on the distance dependence, and therefore typically do not address NOEs between atoms with relatively short covalent bond separations. For these atom pairs, the NOE will be primarily modulated by dynamics in the orientation of the internuclear vector, as it is for backbone amide heteronuclear NOEs. Because the KE approach can rigorously account for both aspects, we have included these low bond separation NOEs in our analysis. As shown in Fig. 4d, which compares experimental intensities to those calculated using a single 2 ns timescale, nearly all atom pairs where the NOE intensity is highly overestimated by the model are separated by 2–5 bonds. This suggests that the orientational motion of the atom pairs is too low and/or the timescale is too slow, both of which result in reduced attenuation of the dipolar interactions. Conversely, the majority of atom pairs having highly underestimated NOE intensities come from those separated by six or more bonds. However, there are a few examples of atoms pairs separated by 2–5 bonds that are predicted to be lower in intensity than actually observed.

One of these, the I13 QG2-HB NOE, is highlighted with an orange line on the right side of Fig. 4d. Upon changing τ_pincer_ from 2 ns to 1 µs, this NOE is most responsible for the better correlation coefficient. This NOE shows a relatively large increase in intensity (orange to green in Fig. 4e) because of differences between the overall motion of the I13 side chain and the motion when open or closed. While the chi 1 angle of I13 shows a relatively equal distribution of the 60° and 300° rotamers, the open or closed conformations are more skewed towards one rotamer or the other. (Fig. 4g)

This population shuffling (Smith et al. 2015) of rotamer conformations makes the CB-CG2 order parameters for both the open and closed conformations higher than the overall order parameter (Fig. 4h). In the EROS3 ensemble, the motion of the I13 side chain is thus distinctly hierarchical, showing differences in both average structure and dynamics depending on whether it is open or closed. The CB-CG2 heavy atom order parameter is likely a good proxy for the order parameter between the beta hydrogen and the dynamically averaged position of the gamma 2 methyl group hydrogens. When τ_pincer_ ≫ τ_c_, the order parameter that determines the NOE rate is the population weighted mean order parameter of the open and closed states, which is much higher than overall order parameter. This helps explain why increasing τ_pincer_ almost three orders of magnitude results in a greater increase in NOE intensity for this pair than others (e.g. Fig. 4f). The difference in fast timescale mean order parameters (determining the NOE rate) and overall order parameters is largely in agreement with previously determined Lipari-Szabo (Lee et al. 1999) and RDC (Fares et al. 2009) order parameters, with the underlying RDC data being used in refinement of the EROS3 ensemble. Though the RDC order parameter is consistent with the ensemble, the experimentally determined Lipari-Szabo parameter is greater than the mean order parameter derived from the EROS3 ensemble. This discrepancy is mirrored in the NOE data, with the experimental buildup curve still exceeding the calculated one. The most straightforward ways to improve the match between the EROS3 ensemble and both datasets would be to either increase the population of the closed conformation or decrease the population of the 60° rotamer in the open conformation.

While increasing τ_pincer_ and decreasing τ_ensemble_ led to a net increase in the NOE intensities for I13 QG2-HB NOE, a net decrease in NOE intensities was also observed for many other pairs. The pair that caused the greatest improvement in correlation after reoptimization of τ_ensemble_ was K11 QD-QE. This pair was already highly dynamic, with the heavy atom CD-CE order parameter being 0.13. Because the mean open/closed order parameter was not much higher (0.15), increasing τ_pincer_ only produced a very small increase in observed NOE intensity. Reoptimization of τ_ensemble_ from 2.0 to 1.4 ns caused a sizable reduction in NOE intensity below the original prediction. However, there is still a substantial overestimation of the NOE rate for not only K11 QD-QE, but also several other NOEs in the lysine side chain including QD-HG2 and QE-HG2. To improve the model, the kinetics for the side chain could be made much faster. Alternatively, the side chain order parameters could be decreased by increasing the overall mobility or again increasing the population of the closed state. Because the pincer mode was the largest amplitude motion and therefore the most likely to have a detectable kinetic dependence, we did not explore adding additional timescales for lower amplitude motions such as the ubiquitin peptide flip (Smith et al. 2016), but instead turned to changing the composition of the ensemble itself.

### Ensemble subselection produces better fits to NOE data

**Fig. 5:**
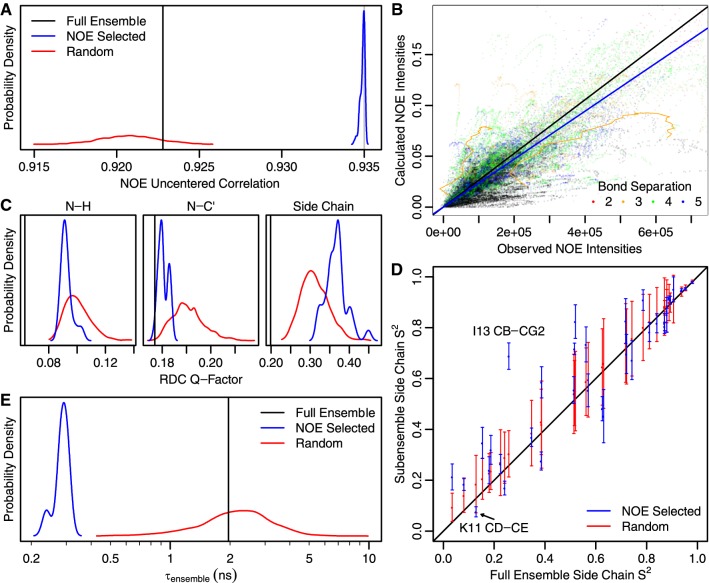
NOE selected subensembles outperform random subensembles. **a** 100 NOE selected subensembles (blue) produce better uncentered correlations (R_u_) than the full ensemble (black), while randomly selected subensembles (red) fit less well than the full ensemble. An R_u_ cutoff of 0.935 (gray line) was used to pick the top 31 scoring ensembles shown in subsequent panels. **b** NOE data for the best selected subensemble. The best scoring subensemble shows slightly less overall magnetization transfer. The blue (NOE selected) and black (full ensemble, Fig. 4d) lines have slope RMS_calc_/RMS_obs_. **c** Cross-validation with RDC data (lower Q-factors are better) shows that the random subensembles match the data less well than the full ensemble. However, ensembles selected by NOEs have better backbone N–H (dashed) and N–C′ (solid) RDC Q-factors than the random subensembles, while the side chain data (dotted) is worse. **d** Part of the decreased side chain performance likely comes from significant changes to overall side chain order parameters, with notable rigidification in a number of residues. Error bars indicate standard deviations. **e** Those side chains are likely rigidified to counteract an almost tenfold decrease in the ensemble timescale

In addition to demonstrating that adding another timescale improved agreement with the NOE data, we also tested how much selecting a refined subset of ensemble members would improve goodness of fit. To do so, we ran a subselection optimization algorithm 100 times starting from different random subsets of the EROS3 ensemble (see “Methods”). The algorithm produced 13–18 member subensembles and consistently improved correlation with the NOE data over the full ensemble (Fig. 5a). By contrast, if random subensembles having same size distribution were generated, the correlations tended to be worse than the full ensemble. This decrease for the random subensembles may stem from the way the EROS3 ensemble was produced, in which 8-member ensembles were collectively fit to NOE/RDC data, each therefore containing a distribution of states that together matched the data. By construction, the full 176-member ensemble would also show a similar overall distribution. However, the generation of small random subsets could very likely miss important states, resulting in lower correlations.

The best scoring subensemble (R_u_ = 0.935) was found two out of 100 times by the algorithm and showed slightly lower fractional magnetization transfer than the full ensemble (Fig. 5c). To determine whether the NOE optimized subensembles were truly better than random subensembles of the same size, we cross-validated with the same RDC dataset that was used to produce the EROS3 ensemble (Fig. 5c). Similar to what was observed for the NOEs (Fig. 5a), the random subensembles showed consistently worse reproduction of the RDCs than the full ensemble. However, the NOE selected subensembles showed better Q-factors for backbone RDCs than the random subensembles, indicating that application of the Kinetic Ensemble approach better preserved the orientation/dynamics of backbone bond vectors. Interestingly, the side chain RDCs showed the opposite trend, with the NOE-selected subensembles being worse than those that were randomly selected.

To determine why that might be the case, we examined how use of the NOE data influenced the ensemble side chain order parameters. We found that the 34 side chain methyls for which RDC data was available showed a number of large deviations from the full ensemble order parameters, particularly towards rigidification (Fig. 5e). Notably, the overall order parameter for I13 CB-CG2 increased from 0.25 (consistent with the RDC S^2^, Fig. 4h) to 0.69 (slightly above the Lipari-Szabo S^2^). While that improves agreement with the NOE data, it worsens RDC reproduction for this methyl. By contrast, in the τ_ensemble_/τ_pincer_ model described above, where differing flexibility at two timescales was allowed, it was possible to fit both RDC and Lipari-Szabo/NOE data simultaneously.

Given the difference between models with hierarchical and non-hierarchical kinetics, we also examined the τ_ensemble_ values derived from the different subensembles (Fig. 5f). While the random subensembles showed a broad distribution of timescales roughly centered on the full ensemble, the NOE selected subensembles had a much narrower distribution almost an order of magnitude faster. We previously hypothesized (Fig. 4h, i) that such faster kinetics and/or reduction in S^2^ would improve the fits to the NOE data for K11 QD-QE as well as many of the other NOEs coming from short bond separations. The NOE selected S^2^ for K11 QD-QE was also lower than the full ensemble (also shown in Fig. 5e), supporting the idea that changes in both τ_ensemble_ and S^2^ could play a role. However, because dynamics of the entire structure are forced to be on the same timescale, other parts are rigidified (like I13), possibly making the ensemble less representative of the overall flexibility in those regions. This highlights not only the exquisite sensitivity of NOEs to both protein flexibility and kinetics, which hasn’t been possible to analyze with previous theoretical approaches, but also the need to make further refinements to the kinetic schemes employed here to better interpret existing NOE datasets.

### Goodness of fit to NOE data is highly correlated with crystal structure resolution

**Fig. 6:**
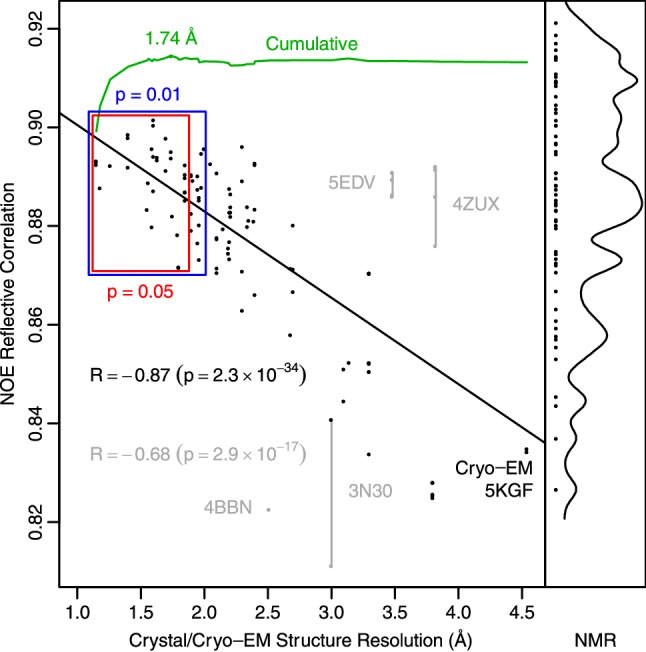
NOE data agreement is highly correlated with crystal structure resolution. 117 ubiquitin domains from 65 different crystal/cryo-EM structures were used to determine their agreement with the NOE buildup data. Across the full range of resolutions (1.15–4.54 Å), the Pearson correlation coefficient between the resolution and agreement with NOE data is − 0.68. Several structures show unexpectedly high correlations (likely because of molecular replacement into a low-resolution map), or low correlation (because of unusual ubiquitin conformations or poor refinement). When the gray points are removed, the Pearson correlation improves to − 0.87. Both Pearson correlation coefficients are statistically significant (p < 10^−16^). The Pearson correlation continues to be statistically significant for structures less than 2 Å (p = 0.01, blue) or 1.85 Å (p = 0.05, red), suggesting that NOE data contains higher resolution data than previously thought. When the crystal structures are combined to create a more dynamic ensemble, starting with those at the best resolution, the agreement increases (green line) and reaches a maximum (green point) when all structures 1.74 Å and better are included. This crystal ensemble approaches the best published NMR ensembles to-date in fitting to the NOE data

Ubiquitin has been extensively studied by both X-ray crystallography and NMR. To test the ability for the Kinetic Ensemble method to discern structural quality, we used it to compute buildup curves for 176 distinct full-length ubiquitin domain structures, including 115 X-ray crystal, 57 solution NMR, 2 solid-state NMR, and 2 cryo-EM structures (from 64, 47, 2, and 1 PDB entries, respectively). Where multiple alternate conformations existed in the PDB, they were treated as different ensemble members. For NMR structures, all ensemble members were included in the calculations. For this analysis we used crystal/cryo-EM structure resolution as a proxy for quality. In that regard, the goodness of fit to the NOE data showed a surprisingly high correlation with structural quality (Fig. 6).

Several PDB entries showed much better agreement with the NOE data relative to their resolution [5EDV (Lechtenberg et al. 2016) and 4ZUX (Morgan et al. 2016)]. The ubiquitin domains in these macromolecular complexes were solved via molecular replacement using crystal structures of ubiquitin with higher resolution, likely producing better coordinates than would otherwise have been possible. Similarly, several structures showing lower agreement with the NOE data also had more than half of their PDB quality metrics lower than the median values for other structures of similar resolution. When these structures are excluded the correlation coefficient improves to − 0.87.

While discrimination of structural quality over resolutions ranging 1.15–4.5 Å using NOE data could be anticipated, it was much more unexpected to find that significant correlations extended down to resolutions ranging 1.15–1.85 Å (p = 0.05) or 1.15–1.98 Å (p = 0.01). This indicates that NOEs likely have higher resolving capability than was previously appreciated. This high correlation was found despite all the potential confounding factors such as contacts within the crystal lattice, the presence of binding partners, covalent linkages, and differences between the cryogenic temperatures where most of the crystal structures were solved and 308 K where the NOEs were determined. A similar analysis with RDC data yields lower correlations, both quantitatively and visually (Fig. S5). This suggests that NOE data is capable of not only ensemble determination, but also distinguishing high-quality average ground-state structures from those with slightly less quality, which is useful in high-resolution structural refinement.

Despite the clear correlation between NOE data and crystal structure resolution, NMR ensembles still fit the NOE data better than the crystal structures. The top scoring NMR models included single structures optimized against NOEs, RDCs, and J-couplings (1D3Z (Cornilescu et al. 1998), and 2MJB (Maltsev et al. 2014)) as well as ensembles fit against multiple types of NMR data including NOEs (Cornilescu et al. 1998; Lange et al. 2008; Fenwick et al. 2011; Maltsev et al. 2014; Lindorff-Larsen et al. 2005; Richter et al. 2007), RDCs (Cornilescu et al. 1998; Lange et al. 2008; Fenwick et al. 2011; Montalvao et al. 2012; Maltsev et al. 2014), Lipari-Szabo order parameters (Lindorff-Larsen et al. 2005; Richter et al. 2007), and scalar couplings (Cornilescu et al. 1998; Maltsev et al. 2014). (Table 1) Those ensembles included ERNST (Fenwick et al. 2011) (2KOX), MUMO (Richter et al. 2007) (2NR2), DER (Lindorff-Larsen et al. 2005) (1XQQ), EROS (Lange et al. 2008) (2K39), and 2LJ5 (Montalvao et al. 2012). However, combining the 22 ubiquitin domains coming from crystal structures with resolutions 1.15-1.74 Å (Table S1) produces an ensemble whose correlation (R_u_ = 0.915) exceeds all but four of the NMR ensembles (EROS3, ERNST, MUMO, and DER).

**Table 1:** Top scoring NMR ensembles

R_u_	PDB ID	Chain	Models	Ligand	Derived from	Year	PDB First Author
0.921	6V5D	A	176			2019	de Groot
0.919	2KOX	A	640			2009	Salvatella (Fenwick et al. 2011)
0.917	2NR2	A	144			2006	Vendruscolo (Richter et al. 2007)
0.916	1XQQ	A	128			2004	Dobson (Lindorff-Larsen et al. 2005)
0.914	2RR9	B	20	tUIMs	Unknown	2010	Shirakawa
0.914	2RR9	A	20	tUIMs	Unknown	2010	Shirakawa
0.914	2K39	A	116			2008	de Groot (Lange et al. 2008)
0.911	2LVQ	B	24	gp78CUE	1D3Z	2012	Das (Liu et al. 2012)
0.910	2LVQ	A	24	gp78CUE	1D3Z	2012	Das (Liu et al. 2012)
0.909	2LVO	A	20	gp78CUE	1D3Z	2012	Das (Liu et al. 2012)
0.909	2LJ5	A	301			2011	Vendruscolo (Montalvao et al. 2012)
0.909	2LVP	A	20	gp78CUE	1D3Z	2012	Das (Liu et al. 2012)
0.908	1D3Z	A	10			1999	Bax (Cornilescu et al. 1998)
0.906	2LVP	B	20	gp78CUE	1D3Z	2012	Das (Liu et al. 2012)
0.905	2MJB	A	20			2014	Bax (Maltsev et al. 2014)
0.904	2Z59	B	10	Rpn13	1D3Z	2007	Walters (Schreiner et al. 2008)
0.903	2BGF	B	10	ubiquitin	1D3Z	2004	Bonvin (van Dijk et al. 2005)

## Discussion

Here we have demonstrated a new approach for modeling NOE data which overcomes several shortcomings in the way previous methods have handled protein motion. First, the Kinetic Ensemble approach directly captures the effect that angular motion has on attenuating NOE rates. For protons separated by 2–4 bonds whose interatomic distances are restricted by covalent geometry, this serves as a sensitive probe of angular dynamics, either in the backbone (i.e. between amide and alpha hydrogens), or along side chains. Second, we have shown that the usual assumptions of distance averaging as a power of three or six are only fully valid for motions occurring at least two orders of magnitude slower or faster than the overall tumbling time. In all models we used to fit the experimental data, at least one of the overall exchange timescales was within two orders of magnitude of the tumbling time, indicating that this assumption should be revisited.

As stated above, this study particularly highlights the sensitivity of NOE data to the timescales of angular and through-space motion. The NOE buildup data, when interpreted in the context of a reasonably accurate structural model, is capable of recovering the overall tumbling time with a high degree of accuracy. Furthermore, NOE data can be used to validate hypotheses about the timescales of particular motions, revealing kinetics up to and potentially more than an order of magnitude slower than molecular tumbling.

In the context of structure determination, the quantitative approach used here to directly calculate spectral intensities shows a surprisingly high correspondence between fit to the NOE data and crystal structure resolution. This relationship holds up despite the substantial number of potential confounding factors that make crystal structures deviate from the solution state of a molecule. This suggests that NOE data may contain considerably more information than previously appreciated about the high-precision structural details typically found in the best resolution crystal structures. The Kinetic Ensemble approach could thus be used to help resolve outstanding questions about the similarity between dynamics in solution and other states like a crystal lattice.

When the ubiquitin ensemble was either split into a kinetic hierarchy or trimmed down to a subset of the full ensemble, sizable changes to flexibility and kinetics were observed after Kinetic Ensemble optimization. This practical result is in agreement with what the theory suggests, namely that NOE data is very sensitive to both motional amplitudes and kinetics. This then implies that when interpreting NOE datasets, it is important to correctly model structure, motion, and kinetics. An inaccuracy in any of those three properties will necessarily have an adverse effect on the other two. A NOE-based structural refinement method that ignores any of those properties is making implicit assumptions which could have a sizable impact on the properties it does consider.

One of the most immediately useful applications of the Kinetic Ensemble approach is to augment existing NMR ensemble calculation methods to build more quantitative models of protein structure and motion. Unlike the eNOE approach, only a single mixing time is required, making it applicable to all standard NOE datasets. Starting with a large pool of candidate structures generated using traditional and/or MD-based refinement methods, the Kinetic Ensemble approach can be used to determine optimal subsets (Rangan et al. 2018; Bottaro and Lindorff-Larsen 2018) that collectively reflect not only the structure, but also the dynamics inherent in the NOE data. This approach can also be used to evaluate hypotheses about the relative rates of motions suggested by molecular dynamics simulations.

Furthermore, it is becoming increasingly common to determine structures with a combination of NOE and RDC data. These methods report on motion at different timescales and this study highlights the potential for them to conflict with one another if there is a substantial amount of motion slower than molecular tumbling. If there is sufficient RDC data to extract dynamical order parameters, incorporation of multiple exchange rates provides a means to reconcile the data from these two experiments.

For the quantification of protein dynamics, this approach would be particularly helpful for loop regions, where adjacent protons along the backbone or side chain could be used to directly probe loop flexibility. This would help discern between true dynamics and model uncertainty due to the lower number of medium- and long-range restraints often found in these regions. Furthermore, this could be used to quantify the motion of sidechains that don’t have a methyl group, such polar and charged amino acids critical for catalysis.

Work is currently underway to extend the Kinetic Ensemble approach to allow gradient-based optimization of atomic coordinates in molecular dynamics simulations. Beyond NOE data, the approach could be used to directly model not only the heteronuclear T_1_/T_2_ times used in fitting Lipari-Szabo order parameters, but also ^1^H T_1_/T_2_ times, which are typically more difficult to interpret because they depend so much on the large network of proton dipole–dipole interactions. We recently applied the Kinetic Ensemble approach to structurally interpret solid-state proton relaxation-dispersion data (Rovó et al. 2019), which is also sensitive to the proton interaction network. Furthermore, it could be used to better model other kinetically sensitive phenomena, like cross-correlated relaxation (CCR), or incorporate less timescale dependent data like residual dipolar couplings.

## Methods

### Ubiquitin sample preparation

The cDNA encoding Ubiquitin was expressed in *Escherichia coli* and the purification protocols were adopted as described earlier (Lazar et al. 1997). Isotopically-labeled (^15^N, or ^15^N/^13^C) and unlabeled Ubiquitin were produced following the same protocol. ^15^NH_4_Cl and [^13^C]-glucose (Cambridge Isotope Laboratories) were used as the sole sources of nitrogen and carbon, respectively.

### NMR spectroscopy

The purified ubiquitin samples (^15^N, ^15^N/^13^C, or unlabeled) were used at a concentration of ~ 3 mM in 50 mM sodium phosphate (pH 6.5) containing 100 mM NaCl and 0.05% (w/v) sodium azide. All NMR experiments were carried out at 308 K on a Bruker Avance 900 MHz spectrometer with cryogenic probe. The experiments used for complete resonance assignment were as follows: 3D HNCACB, 3D HCCH-TOCSY, 3D ^15^N-edited NOESY-HSQC, and 3D ^13^C-edited NOESY-HSQC (Bax and Grzesiek 1993). A series of 2D [^1^H-^1^H]-NOESY experiments with mixing times varying from 5 to 500 ms (equally spaced intervals of 15 ms) were recoded with 300 and 1024 complex points along t_1_ and t_2_ dimensions, respectively. The same experiments with NOE mixing times of 80 ms, 155 ms, 215 ms, 305 ms, 410 ms and 500 ms were repeated for error calculation. All NMR data were processed using NMRPipe and analyzed with nmrDraw (Delaglio et al. 1995) and CARA (Keller 2004). CYANA (Güntert and Buchner 2015) was used for stereospecific assignment of 3D NOESY cross peaks. 3D NOE assignments were manually transferred to the 2D experiments and corresponding intensities were determined as a function of NOE mixing time to generate the NOE buildup curves.

### Calculation of correlation functions and spectral densities with kinetic ensemble approach

The Kinetic Ensemble method starts with a matrix of transition rates between structures within an ensemble to directly determine correlation functions and spectral densities, from which NMR observables are calculated. An overview of the slower numerical method and a faster analytical solution is shown in Fig. S1. In a transition rate matrix, *Q*, the rate of a transition from state *i* to state *j* is represented by element *q*_*ij*_, with *i* being the row index and *j* being the column index. The diagonal elements, *q*_*ii*_, are defined ${{q}_{ii}}=-\underset{j\ne i}{\mathop \sum}\,{{q}_{ij}}$. The probabilities, *p*_*ij*_(*τ*), of transitioning from state *i* to state *j* after a given lag time, *τ*, can be calculated using the matrix equation, $P\left(\tau \right)={{e}^{\tau Q}}$. If a system is at a given state (*s*_*i*_) at time *t*, and state (*s*_*j*_) at time *t *+ *τ*, then one can define a correlation function $C\left(\tau \right)=\underset{i,j}{\mathop \sum}\,{{\pi}_{i}}{{p}_{ij}}\left(\tau \right){{c}_{ij}}$, where π_*i*_ is the equilibrium population of a state and *c*_*ij*_ represents the value of a function, *c*(*s*_*i*_,*s*_*j*_), that gives the strength of the correlation between two different states. NMR observables can then be quantified by evaluating *c*(*s*_*i*_,*s*_*j*_) as shown below.

In the case of NOE calculations, *s*_*i*_ is defined by the vector $\overrightarrow {{r_{i} }}$, connecting two nuclei in state *i*. Given scalar *x*_*i*_, *y*_*i*_, and *z*_*i*_ components of that vector and the internuclear distance *r*_*i*_, the cartesian dipole interaction tensor, *D*_*i*_, is defined:

$$D_{i} = \frac{1}{{r_{i}^{5} }}\left( {\begin{array}{*{20}c} {3x_{i}^{2} - r_{i}^{2} } & {3x_{i} y_{i} } & {3x_{i} z_{i} } \\ {3y_{i} x_{i} } & {3y_{i}^{2} - r_{i}^{2} } & {3y_{i} z_{i} } \\ {3z_{i} x_{i} } & {3z_{i} y_{i} } & {3z_{i}^{2} - r_{i}^{2} } \\ \end{array} } \right)$$

The strength of the correlation for two states (dipole–dipole interaction) can then be calculated as follows:

$$c\left( {s_{i} ,s_{j} } \right) = \frac{1}{6}{\text{tr}}\left( {D_{i} D_{j} } \right)$$

To aid in computational efficiency, the *D*_*i*_ tensor can be recast as a vector:

$$\overrightarrow {{d_{i} }} = \frac{1}{{r^{5} }}\left[ {z_{i}^{2} - \frac{1}{2}\left( {x_{i}^{2} + y_{i}^{2} } \right),\frac{1}{\sqrt 3 }\left( {x_{i}^{2} - y_{i}^{2} } \right),\frac{2}{\sqrt 3 }x_{i} z_{i} ,\frac{2}{\sqrt 3 }y_{i} z_{i} ,\frac{2}{\sqrt 3 }x_{i} y_{i} } \right]$$

Such that the normalized trace of the matrix product is equal to the dot product of the vectors:

$$c\left( {s_{i} ,s_{j} } \right) = \frac{1}{6}tr\left( {D_{i} D_{j} } \right) = \overrightarrow {{d_{i} }} \cdot \overrightarrow {{d_{j} }}$$

For the present work, an important property of both the matrix and vector representations of the interaction tensor is that the average value of $\frac{1}{6}{\text{tr}}\left( {D_{i} D_{j} } \right)$ or $\overrightarrow {{d_{i} }} \cdot \overrightarrow {{d_{j} }}$. enumerated over two sets of states is equal to applying the same operation once to the average tensor for each set:

$$\left\langle {\frac{1}{6}tr\left( {D_{i} D_{j} } \right)} \right\rangle = \frac{1}{MN}\mathop {\mathop \sum \limits^{M} }\limits_{i} {\mkern 1mu} \mathop {\mathop \sum \limits^{N} }\limits_{j} {\mkern 1mu} \frac{1}{6}tr\left( {D_{i} D_{j} } \right) = \frac{1}{6}tr\left( {\left\langle {D_{i} } \right\rangle \left\langle {D_{j} } \right\rangle } \right) = \left\langle {\overrightarrow {{d_{i} }} \cdot \overrightarrow {{d_{j} }} } \right\rangle = \left\langle {\overrightarrow {{d_{i} }} } \right\rangle \cdot \left\langle {\overrightarrow {{d_{j} }} } \right\rangle$$

This property, which is a straightforward extension of distributivity, enables much more rapid calculation of the correlation function exponential prefactors. It also makes gradient-based optimization computationally feasible, where the goodness of fit of a whole ensemble to NOE buildup data is differentiated with respect to kinetic or structural parameters.

The transition probability matrix, $P\left( \tau \right) = e^{\tau Q}$, can be calculated by diagonalization of the matrix, $Q = V \, \varLambda \, V^{ - 1}$, where V is a column matrix of the eigenvectors and Λ is a diagonal matrix with the corresponding eigenvalues. The exponential can then be evaluated $P\left( \tau \right) = Ve^{\tau \varLambda } V^{ - 1}$. When incorporated into the internal correlation function, $C_{\text{I}} \left( \tau \right) = \mathop \sum \limits_{i,j} {\mkern 1mu} \pi_{i} p_{ij} \left( \tau \right)c_{ij}$, this produces a multi-exponential decay, $C_{\text{I}} \left( \tau \right) = \mathop \sum \limits_{i} {\mkern 1mu} a_{i} e^{{\lambda_{i} \tau }}$, with decay rates, −λ_i_, coming from the indidual eigenvalues. The exponential prefactors, *a*_*i*_, associated with each rate can be calculated $a_{i} = \mathop \sum \limits_{j} {\mkern 1mu} \pi_{j} v_{ji} \mathop \sum \limits_{k} {\mkern 1mu} v_{ik}^{ - 1} c_{jk}$, with $v_{ji}$ being the element at row *j*, column *i* of $V$. With regularly structured transition rate matrices, as used in this work, the eigenvalues are very often degenerate, and the exponential prefactors can be summed over the eigenvectors associated with a given eigenvalue. If A_λ_ is the square matrix formed by multiplying the columns of *V* by the rows of *V*^−1^ corresponding to the given eigenvalue, and Π is a diagonal matrix containing the equilibrium state populations, then the exponential prefactor can be calculated via the matrix operation, $a_{\lambda } = \left( {\varPi A_{\lambda } } \right) \cdot C$. In that formula, standard matrix multiplication is used with Π and A_λ_, from which the result is combined with C through the dot product operating over the individual matrix elements.

The rate matrices used in this work produce A_λ_ matrices with recognizable patterns (Fig. S2c, g). These can be expressed as a linear combination of matrices, G_i_. The G matrices are structured such that the rows and columns can be reordered to create a block matrix where all rows and columns contain only a single block with nonzero elements. Even without reordering, this is true for all matrices shown in Fig. S2h except for G_3_, which requires reordering. The values of the G matrices are set such that all rows sum to 1. Because the group matrices separate states into blocks, it can be shown that $\left( {\varPi G_{i} } \right) \cdot C = g_{i} = \mathop {\mathop \sum \limits^{{N_{i} }} }\limits_{j = 1} {\mkern 1mu} \frac{{N_{j} }}{N}\left| {\frac{1}{{N_{j} }}\mathop {\mathop \sum \limits^{{N_{j} }} }\limits_{k = 1} {\mkern 1mu} \overrightarrow {{d_{ijk} }} } \right|^{2}$, where *N* is the number of states, *N*_*i*_ is the number of nonzero blocks in matrix G_i,_*N*_*j*_ is the number of states in block *j*, and $\overrightarrow {{d_{ijk} }}$ is tensor *k* of block *j* of group *i*. Examples of this equality are shown in Fig. S2d, h. The exponential prefactors can then be rewritten as follows:

$$a_{\lambda } = \mathop {\mathop \sum \limits^{{N_{G} }} }\limits_{i = 1} {\mkern 1mu} k_{\lambda i} \left( {\varPi G_{i} } \right) \cdot C = \mathop {\mathop \sum \limits^{{N_{G} }} }\limits_{i = 1} {\mkern 1mu} k_{\lambda i} g_{i} = \mathop {\mathop \sum \limits^{{N_{G} }} }\limits_{i = 1} {\mkern 1mu} k_{\lambda i} \mathop {\mathop \sum \limits^{{N_{i} }} }\limits_{j = 1} {\mkern 1mu} \frac{{N_{j} }}{N}\left| {\frac{1}{{N_{j} }}\mathop {\mathop \sum \limits^{{N_{j} }} }\limits_{k = 1} {\mkern 1mu} \overrightarrow {{d_{ijk} }} } \right|^{2}$$

where *N*_*G*_ is the number of group matrices and *k*_*λi*_ is the coefficient associated with a given group matrix (usually a small positive or negative integer). Those coefficients are algorithmically determined from the structure of the rate matrix. While eigendecomposition of the rate matrices and construction of the corresponding A_λ_ and G_i_ matrices can be helpful in determining how this calculation should be conducted and validating that it is done correctly, in a production situation this is unnecessary, and the last expression of the above equation can be evaluated directly. This enables a dramatic speedup in the calculations, as eigendecomposition/matrix multiplication has a running time of approximately O(s^3^) and calculating all *c*_*ij*_ elements is O(s^2^), where s is the number of states in the transition rate matrix (i.e. the number of structures in the ensemble). In contrast, this method has a computational complexity of O(sl), where l is the number of unique eigenvalues. This enables the method to scale to very large ensembles. The largest used in this method is 176 members. As discussed below, for methyl–methyl interactions the ensemble is expanded to 1584 members (9 × 176), which would be computationally prohibitive with O(s^3^) scaling.

Once all a_λ_ coefficients are determined, the spectral density function can be directly evaluated without first determining the correlation function. Taking into account molecular tumbling, assumed to be isotropic, the rotational correlation function is multiplied by the internal correlation function to give the full correlation function $C\left( \tau \right) = e^{{ - \tau /\tau_{c} }} C_{\text{I}} \left( \tau \right) = e^{{ - \tau /\tau_{c} }} \mathop \sum \limits_{i} {\mkern 1mu} a_{i} e^{{\lambda_{i} \tau }}$. This expression can be simplified using modified eigenvalues, $\lambda_{i}^{\prime } = \lambda_{i} - 1/\tau_{c}$, making the final correlation function $C\left( \tau \right) = \mathop \sum \limits_{i} {\mkern 1mu} a_{i} e^{{\lambda_{i}^{\prime } \tau }}$. Fourier transformation of that gives the spectral density function, $J\left(\omega \right)=-2\underset{i}{\mathop \sum}\,{{a}_{i}}\lambda_{i}^{\prime}/\left(\lambda {{_{i}^{\prime}}^{2}}+{{\omega}^{2}} \right)$. By making the substitution, $\lambda_{i}^{\prime } = - 1/\tau_{i}^{\prime }$, that expression can be rearranged to $J\left( \omega \right)=2\underset{i}{\mathop{\sum }}\,{{a}_{i}}\tau_{i}^{\prime }/\left( 1+{{\omega }^{2}}\tau_{i}^{\prime 2} \right)$, which is a generalization of the extended model free spectral density. In the regime where J(0) dominates, $J\left( 0 \right) = 2\mathop \sum \limits_{i} {\mkern 1mu} a_{i} \tau_{i}^{\prime }$, and the NOE cross relaxation rate is directly proportional to the time constants ($\tau_{i}^{\prime}$) associated with molecular motions and the *a*_*i*_ coefficients. One of the key advancements made in tinetic Ensemble method is an efficient analytical method to rigorously calculate $J\left(\omega \right)$ from a temporally partitioned structural ensemble. The magnitude of the coefficients (${{a}_{i}}$) are determined by the extent of additional orientational dynamics arising from motions at a given timescale, convoluted with simultaneous changes in internuclear distances.

### Methyl and aromatic group rotation

For computational simplicity, many structural modeling programs do not explicitly sample symmetrical hydrogen arrangements on methyl or aromatic groups. However, these structural rearrangements can play a significant role in observed NOE data. These motions can be addressed through either explicit modeling of the rotations or the use of pseudoatoms (Koning et al. 1990; Liu et al. 1992). In this work, we enumerate these rotations by expanding the ensemble to include relabeled methyl and aromatic group atoms and using an expanded transition rate matrix. For a methyl group, the algorithm does the equivalent of tripling the number of ensemble members and then relabeling the methyl atoms such that they hop between three possible conformations. The kinetics of the rotations are modelled using 2 × 2 (aromatic) or 3 × 3 (methyl) transition rate matrices, Q_r_, in which individual transition rates are set to 1/(2τ_aromatic_) or 1/(3τ_methyl_), respectively. Because the rotations are treated as being kinetically independent from other protein motions, the overall transition rate matrix, Q_o_, is calculated using the Kronecker sum of the input and rotation rate matrices, Q_o_ = Q ⊕ Q_r_. Interactions between atoms on two different rotation groups are handled by additional enumeration. In the present work τ_aromatic_ was fixed at 100 µs and τ_methyl_ was fixed at 1 ps. The correlation with NOE data plateaus around those values (Fig. S3), which are also consistent with previously determined timescales of 1 µs–100 ms for aromatic groups (Weininger et al. 2014) and 1–100 ps for methyl groups (Xue et al. 2007).

### Calculating spectral intensities

Where necessary, input structures and ensembles were protonated with Reduce (Word et al. 1999). No pseudoatoms were used in the calculations. Spectral densities for all proton dipole–dipole interactions were calculated as described above, including those between equivalent atoms in methyl/aromatic groups. Using those spectral densities, the relaxation matrix, R, was calculated using the well-established Solomon equations (Brüschweiler and Case 1994). Fractional magnetization transfer between protons for a given mixing time, τ_m_, was calculated using the matrix exponential ${{e}^{{{t}_{m}}R}}$. Theoretical NOE intensities between sets of indistinguishable nuclei were determined by summing blocks of the resulting matrix. Where applicable in the Results section, we refer to this summed fractional magnetization transfer using pseudoatom (Q) notation. For crystal structures where there were multiple alternate conformations of atoms available, separate models were created corresponding to each of the alternate conformations, and the structure was scored as an ensemble.

### Evaluating goodness of fit

NOE intensities calculated using the Kinetic Ensemble method are typically expressed as fractional magnetization transfer from one nucleus to another. Because the appropriate scaling factor between those values and the experimentally measured NOE intensities is not known a priori, we use an uncentered correlation coefficient, ${{R}_{\text{u}}}$, to determine the goodness of fit. In the uncentered form, the mean values of each set of data are set to zero such that the formula reduces to ${{R}_{\text{u}}}=xy/\sqrt{{{x}^{2}}{{y}^{2}}}$. This has the desired effect of penalizing deviations from a zero y-intercept. However, it is important to note that the uncentered correlation coefficient is higher than the ordinary Pearson correlation coefficient.

### Optimization of timescales

Once the correlation function exponential prefactors (${{a}_{i}}$) have been calculated for all of the dipole–dipole interactions in a given ensemble, the overall exchange rates (${{k}_{i}}$) can be optimized independently of those prefactors. Depending on how a rate matrix is constructed, the eigenvalues (${{\lambda}_{i}}$) represent either a single exchange timescale or multiple exchange timescales. For instance, in Fig. S2a, b, the unique eigenvalues each reflect distinct rates, with $\lambda_{1} = - \;k_{\text{s}}$ and $\lambda_{2} = \lambda_{3} = - \;k_{\text{f}}$. However, in Fig. S2e, f, one of the unique eigenvalues represents the sum of the exchange rates, with $\lambda_{3} = - \;(k_{\text{s}} + k_{\text{f}} )$. This happens when the rate matrix is constructed from the Kronecker sum of other rate matrices, as is done for methyl and aromatic group rotation.

Optimization of kinetic coordinates requires determining the gradient of the goodness of fit with respect to the coordinates, $\partial {{R}_{\text{u}}}/\partial {{\tau}_{i}}$ in the case of the timescales. The most computationally challenging aspect of that calculation is the derivative of $e^{{\tau_{m} R}}$, where $\tau_{m}$ is the NOE mixing time and *R* is the dipole–dipole relaxation matrix (Yip and Case 1989). The computation of this derivative has been previously described (Yip and Case 1989; Jennrich and Bright 1976; Kalbfleisch and Lawless 1985), and involves diagonalization of *R* and the determination of the derivative of individual elements of *R* with respect to either the kinetic coordinates. The currently implemented version of the algorithm uses the earlier matrix form of the derivative (Jennrich and Bright 1976; Kalbfleisch and Lawless 1985), which does not take advantage of the sparsity of detectable NOE cross peaks that can significantly reduce the computational cost (Yip and Case 1989). That optimization will be incorporated in future versions of the algorithm. Using $R_{\text{u}} \left( {\tau_{i} } \right)$ and $\partial R_{\text{u}} /\partial \tau_{i}$, the timescales are then numerically optimized with the L-BFGS-B algorithm.

### Current assumptions

In its current form, the Kinetic Ensemble approach assumes the following have a negligible effect: non-uniform initial magnetization, anisotropic molecular tumbling, dependence of molecular tumbling on internal motions, and solvent hydrogen exchange (e.g. for amide or hydroxyl-protons). The treatment of many of these will be addressed in future work. In that regard, adapting complementary ideas from the eNOE approach to determine initial magnetization would be particularly helpful.

### Ensemble subselection

Subensembles were selected from the EROS3 ensemble starting from random ensembles in which each member was either included or excluded with an even probability, resulting in an initial size of approximately 88 members. In successive rounds each of the 176 members had its status as included or excluded swapped one at a time, after which τ_c_ and τ_ensemble_ were optimized using gradient based minimization from their previously optimal values. After evaluating the 176 swaps, the one resulting in the greatest improvement in R_u_ was accepted. Further rounds of swaps were attempted in the same way until swapping no longer improved the fit to experimental data. To construct the random reference ensembles shown in red on Fig. 5, random subensembles were chosen with the same distribution of sizes as the NOE selected ensembles.

## Electronic supplementary material

Below is the link to the electronic supplementary material.

Supplementary material 1 (PDF 763 kb)
