# Supplementary material for: Enhancing NMR derived ensembles with kinetics on multiple timescales
Source: J Biomol NMR. 2019 Dec 14;74(1):27–43. doi: 10.1007/s10858-019-00288-8 (PMC7015964; doi:10.1007/s10858-019-00288-8)
Supplement: Supplementary file 1 — Supplementary material 1 (PDF 763 kb) [file 10858_2019_288_MOESM1_ESM.pdf]

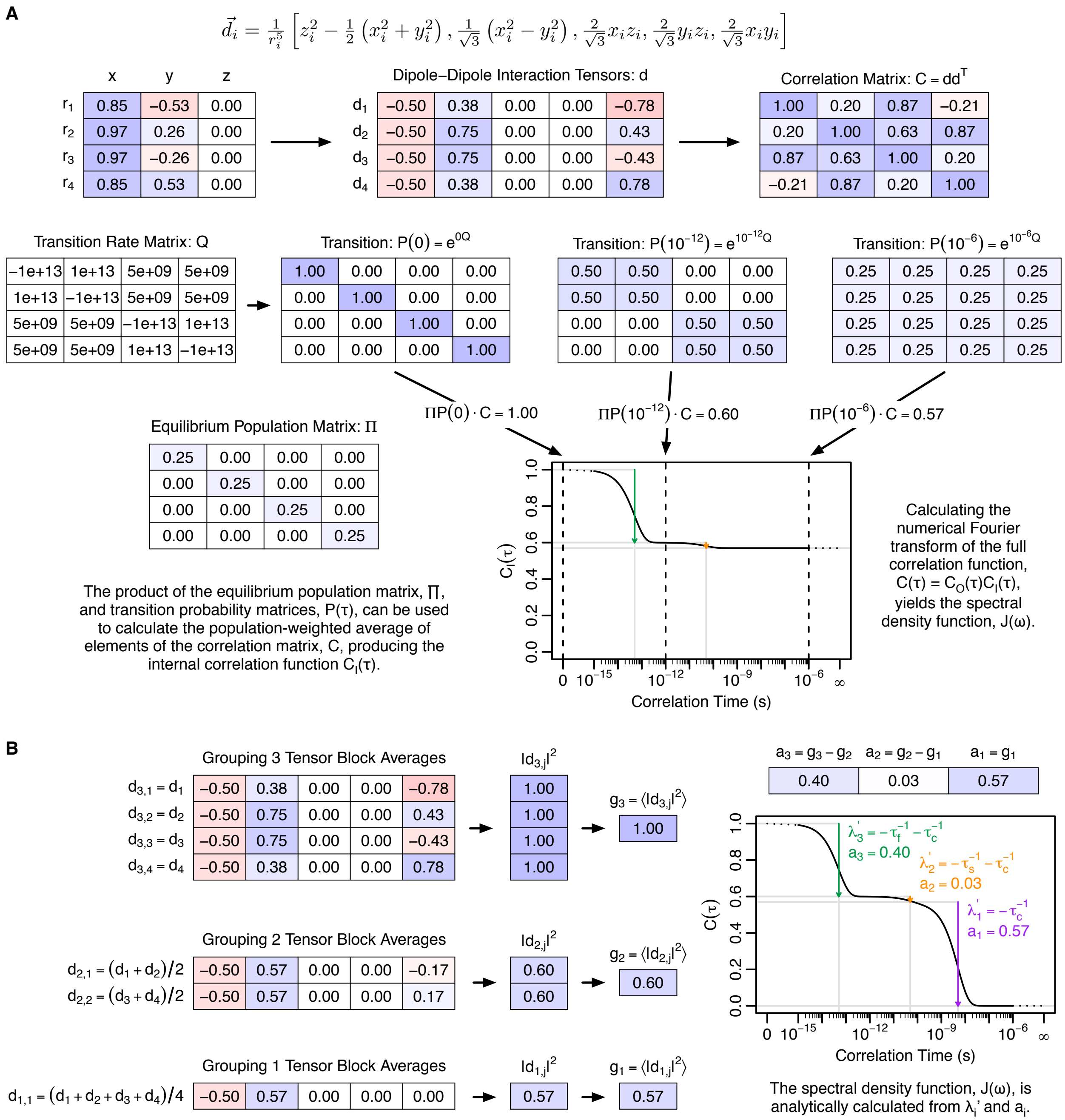

Figure S1. Analytical spectral density calculation involves far fewer operations than numerical solution

Numerical and analytical solutions for the correlation and spectral density functions shown in Figure 1 are outlined. **A)** Numerical solution: From the internuclear vectors (r_i) for a given proton pair, the vector forms of the dipole-dipole interaction tensors (d_i) are calculated. The matrix of correlation values is then created by taking all pairwise dot products of those vectors, here calculated with matrix multiplication. Determining weights for elements of the correlation

matrix requires propagating the transition rate matrix (Q) at different times (τ) to calculate transition probability matrices ($P(\tau)$). Each value of the correlation function is determined by multiplying the equilibrium probability matrix ($\prod$) by the transition probability matrix, followed by taking the element-wise dot product with the correlation matrix. To accurately estimate the spectral density function, the correlation function must be calculated at many values of τ covering the complete decay, including overall tumbling of the molecule. With typically log spaced τ, the spectral density function can be determined using piecewise numerical integration (i.e. Fourier transformation) of the correlation function. **B)** Analytical solution: Following calculation of d_i as in **A**, blocks of kinetically related tensors are averaged. Groupings depend on the factoring of matrices (A_i) derived from eigenvectors of Q into block matrices (G_i), as shown in Figure S2. The self-dot products ($d_{i,j} \cdot d_{i,j}$, equal to the Euclidean norm squared, $|d_{i,j}|^2$) of the averaged tensors are then calculated, followed by further averaging over the groups ($g_i = \langle |d_{i,j}|^2 \rangle$). The correlation function can then be analytically described using a multiexponential decay with the exponential prefactors (a_i) calculated from linear combinations of g_i, along with decay rates (λ_i') determined from eigenvalues of Q. The spectral density function follows directly from analytical Fourier transformation of that formula. This involves several orders of magnitude fewer operations than the numerical method.

A Q

−6	4	1	1
4	−6	1	1
1	1	−6	4
1	1	4	−6

B $V:\ \lambda = 0, -4, -10, -10$

−0.50	0.50	0.71	0.00
−0.50	0.50	−0.71	0.00
−0.50	−0.50	−0.00	−0.71
−0.50	−0.50	−0.00	0.71

C $A_0 = V_{1:4,1} \times V^{-1}_{1,1:4} = G_1$

0.25	0.25	0.25	0.25
0.25	0.25	0.25	0.25
0.25	0.25	0.25	0.25
0.25	0.25	0.25	0.25

$A_{-4} = V_{1:4,2} \times V^{-1}_{2,1:4} = -G_1 + G_2$

0.25	0.25	−0.25	−0.25
0.25	0.25	−0.25	−0.25
−0.25	−0.25	0.25	0.25
−0.25	−0.25	0.25	0.25

$A_{-10} = V_{1:4,3:4} \times V^{-1}_{3:4,1:4} = -G_2 + G_3$

0.5	−0.5	0	0
−0.5	0.5	0	0
0	0	0.5	−0.5
0	0	−0.5	0.5

D $G_1:\ \Pi G_1 \cdot C = |(d_1 + d_2 + d_3 + d_4)/4|^2$

0.25	0.25	0.25	0.25
0.25	0.25	0.25	0.25
0.25	0.25	0.25	0.25
0.25	0.25	0.25	0.25

$G_2:\ \Pi G_2 \cdot C = (|(d_1 + d_2)/2|^2 + |(d_3 + d_4)/2|^2)/2$

0.5	0.5	0	0
0.5	0.5	0	0
0	0	0.5	0.5
0	0	0.5	0.5

$G_3:\ \Pi G_3 \cdot C = (|d_1|^2 + |d_2|^2 + |d_3|^2 + |d_4|^2)/4$

1	0	0	0
0	1	0	0
0	0	1	0
0	0	0	1

E Q

−7	5	2	0
5	−7	0	2
2	0	−7	5
0	2	5	−7

F $V:\ \lambda = 0, -4, -10, -14$

−0.50	0.50	0.50	0.50
−0.50	0.50	−0.50	−0.50
−0.50	−0.50	0.50	−0.50
−0.50	−0.50	−0.50	0.50

G $A_0 = V_{1:4,1} \times V^{-1}_{1,1:4} = G_1$

0.25	0.25	0.25	0.25
0.25	0.25	0.25	0.25
0.25	0.25	0.25	0.25
0.25	0.25	0.25	0.25

$A_{-4} = V_{1:4,2} \times V^{-1}_{2,1:4} = -G_1 + G_2$

0.25	0.25	−0.25	−0.25
0.25	0.25	−0.25	−0.25
−0.25	−0.25	0.25	0.25
−0.25	−0.25	0.25	0.25

$A_{-10} = V_{1:4,3} \times V^{-1}_{3,1:4} = -G_1 + G_3$

0.25	−0.25	0.25	−0.25
−0.25	0.25	−0.25	0.25
0.25	−0.25	0.25	−0.25
−0.25	0.25	−0.25	0.25

$A_{-14} = V_{1:4,4} \times V^{-1}_{4,1:4} = G_1 - G_2 - G_3 + G_4$

0.25	−0.25	−0.25	0.25
−0.25	0.25	0.25	−0.25
−0.25	0.25	0.25	−0.25
0.25	−0.25	−0.25	0.25

H $G_1:\ \Pi G_1 \cdot C = |(d_1 + d_2 + d_3 + d_4)/4|^2$

0.25	0.25	0.25	0.25
0.25	0.25	0.25	0.25
0.25	0.25	0.25	0.25
0.25	0.25	0.25	0.25

$G_2:\ \Pi G_2 \cdot C = (|(d_1 + d_2)/2|^2 + |(d_3 + d_4)/2|^2)/2$

0.5	0.5	0	0
0.5	0.5	0	0
0	0	0.5	0.5
0	0	0.5	0.5

$G_3:\ \Pi G_3 \cdot C = (|(d_1 + d_3)/2|^2 + |(d_2 + d_4)/2|^2)/2$

0.5	0	0.5	0
0	0.5	0	0.5
0.5	0	0.5	0
0	0.5	0	0.5

$G_4:\ \Pi G_4 \cdot C = (|d_1|^2 + |d_2|^2 + |d_3|^2 + |d_4|^2)/4$

1	0	0	0
0	1	0	0
0	0	1	0
0	0	0	1

Figure S2. Eigenvector analysis of rate matrices enables decomposition into a linear combination of state subsets

A) A hierarchical rate matrix is constructed similar to Figure 1B, with $k_f = 10$ and $k_s = 4$. Inter-macrostate rates are set to $k_s/4$ and intra-macrostate rates are set to $(k_f - (1\text{-}2/4)k_s)/2$. **B)** Decomposition of the matrix into eigenvalues (λ) and a matrix of eigenvectors (V) yields three unique eigenvalues. **C)** For each eigenvalue, the product of the columns of V and the rows of V^{-1} corresponding to that eigenvalue yields the eigenvector subset product matrix (A_λ) that can be used to weight the matrix (C) of dipole-dipole correlations between two states. The correlations must also be weighted by the equilibrium populations of a given state, which are given by the diagonal of A_0. Applying both weightings and summing over the elements of C gives the pre-exponential factor in the correlation function associated with a given eigenvalue. The eigenvector subset product matrices ($V \times V^{-1}$) can be expressed as a linear combination of group matrices. **D)** Group matrices are defined such that $(\Pi G_i) \cdot C$ is equivalent to various schemes of averaging the modulus squared of the internuclear vector tensors, $\vec{d_i}$. When r_i is fixed $|\vec{d_i}|^2 = r_i^{-6} S_i^2$. Therefore, drawing analogy to extended model free formalism, $(\Pi G_1) \cdot C$ is equivalent to $r^{-6} S^2$ and $(\Pi G_2) \cdot C$ is equivalent to $r^{-6} S_f^2$. **E)** A similar non-hierarchical rate matrix was

constructed by taking the Kronecker sum ($Q = Q_f \oplus Q_s$) of a pair of two state matrices, one (Q_f) with interstate rates of $k_f/2$ and another (Q_s) with interstate rates of $k_s/2$. **F)** This matrix results in an additional eigenvalue that is the sum of $-k_f$ and $-k_s$. **G/H)** The $\lambda = -14$ eigenvector subset product matrix is a linear combination of all four group matrices.

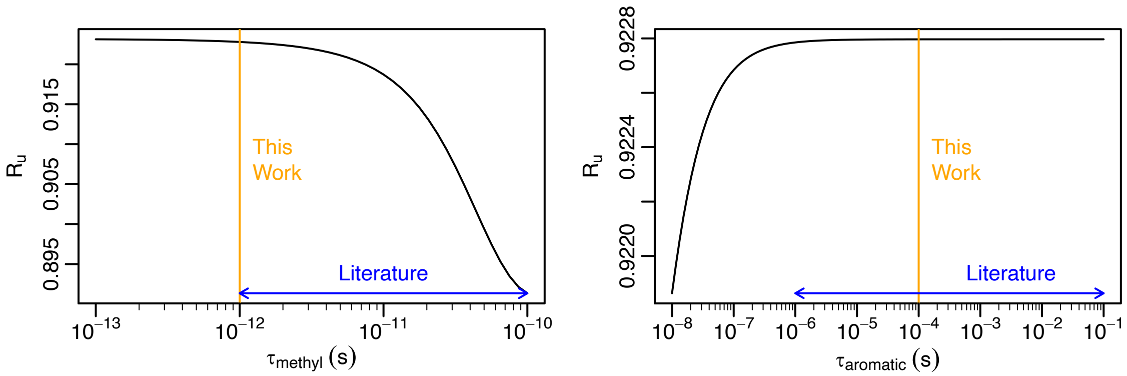

Figure S3. Rotation timescales used in this work fall in correlation plateau region and are consistent with literature values.

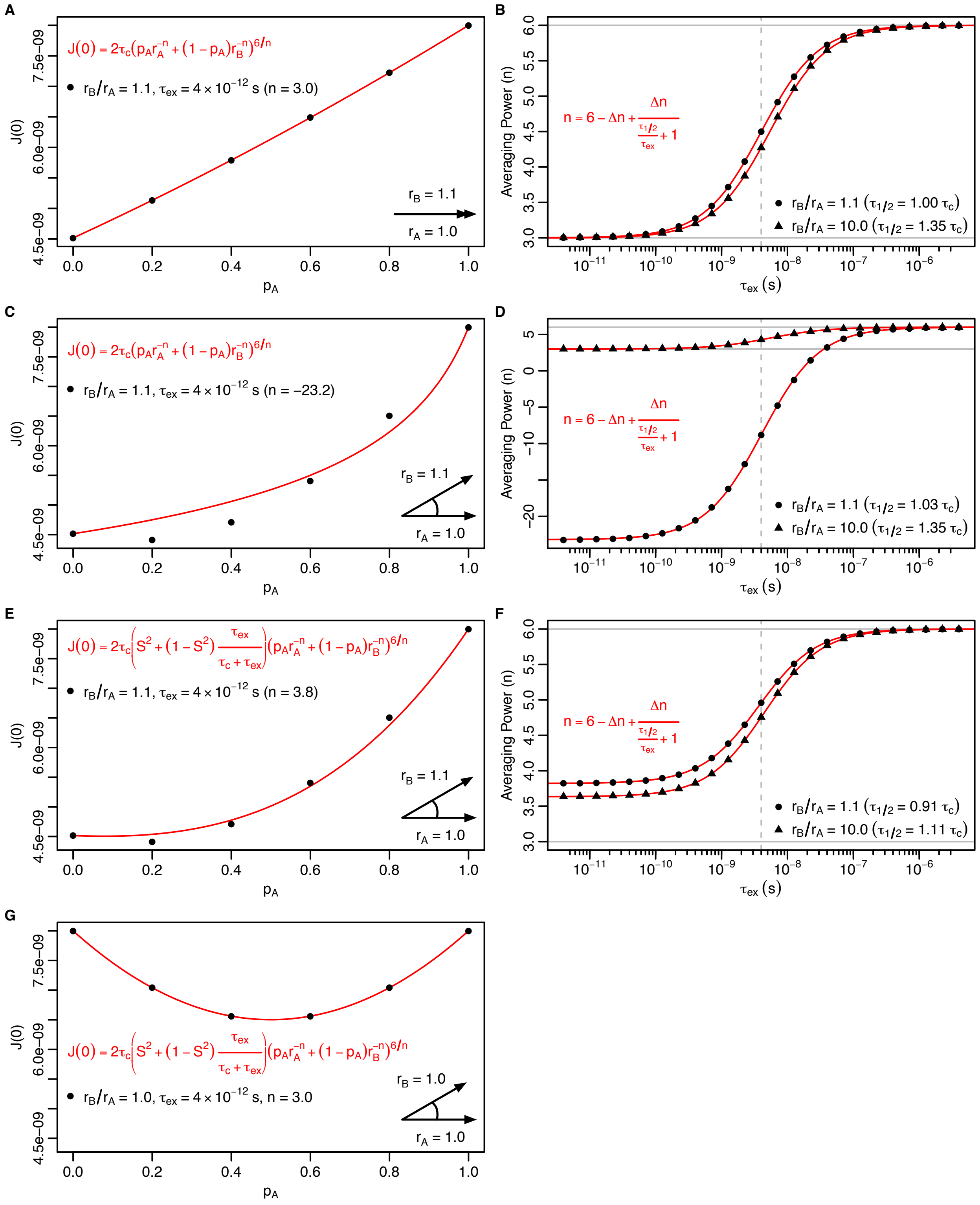

Figure S4. Simultaneous changes in distance and angle are not modeled well by simple analytical expressions

A) An example of the Kinetic Ensemble modeling (black points) and corresponding fit (red line) used to create Figure 2, in this case with parallel internuclear vectors having $r_A = 1.0$ and $r_B = 1.1$. A fit to the equation shown in red (with free parameter n) yields one data point shown in Figure 2. **B)** The data shown in Figure 2 was refit with an additional free parameter, Δn. **C)** If a 30° angle is introduced in between the internuclear vectors, a fit with the same equation as in **A** leaves large systematic residuals. **D)** For small distance ratios, the resulting averaging powers go to extreme values in a futile attempt to fit the data. **E)** Adding a model free-like order parameter correction factor to the fitting equation improves the fit but still fails to model the behavior correctly. **F)** The resulting averaging powers continue to be distorted at fast exchange timescales in an attempt to fit the correlated changes in distance and orientation. **G)** If equidistant internuclear vectors are used, the order parameter correction factor fully captures the changes in populations. Because distances were equal, the averaging power was set to three and the red equation was directly evaluated from the input parameters.

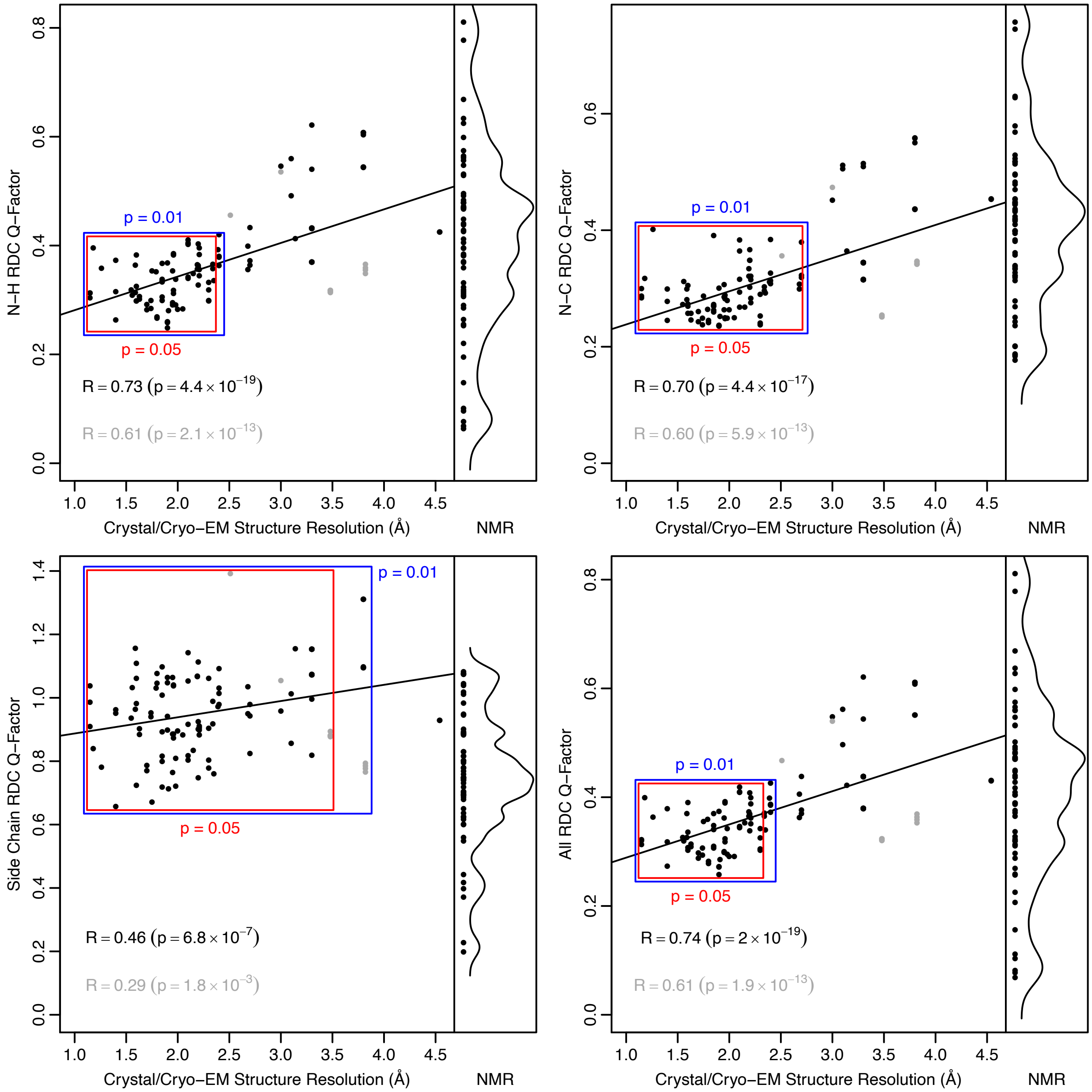

Figure S5. Crystal resolution is less correlated with RDCs especially at high resolution

Theses plots are produced in the same manner as Figure 6. The four plots show Q factors for N-H, N-C', side chain, and all RDCs pooled together. The RDCs used here were the same as those used for refinement and/or cross validation of the EROS ensembles[11]. The alignment tensors were fit simultaneously for all RDC types and all Q factors were derived from the same alignment tensor.

R_u	Resolution	PDB ID	# Alt.	Year	R_u	Resolution	PDB ID	# Alt.	Year
0.902	1.6	3M3J:A	2	2010	0.889	3.48	5EDV:F	1	2015
0.901	1.6	3M3J:E	2	2010	0.889	1.91	5D0M:B	1	2015
0.899	1.4	2WWZ:A	2	2009	0.889	2.3	4S22:D	1	2015
0.898	1.4	4M0W:B	2	2013	0.889	1.56	4LJO:B	1	2013
0.898	1.7	3VUX:A	2	2012	0.888	1.98	3VUY:C	1	2012
0.896	2.3	4S22:B	1	2015	0.888	1.18	3A9J:B	2	2009
0.896	1.59	5GOI:B	1	2016	0.888	2.2	3A33:B	1	2009
0.896	2	4WLR:C	1	2014	0.887	1.95	4MSQ:B	2	2013
0.895	1.74	4MSM:B	1	2013	0.887	1.6	3M3J:C	2	2010
0.895	1.98	3VUY:A	1	2012	0.887	1.85	3ALB:D	1	2010
0.894	1.6	2ZNV:C	1	2008	0.887	1.85	3ALB:A	1	2010
0.894	1.63	4K1R:D	1	2013	0.887	2.21	5AIU:C	1	2015
0.894	1.74	4MSM:D	1	2013	0.886	3.48	5EDV:E	1	2015
0.893	1.63	4K1R:B	1	2013	0.886	3.48	5EDV:H	1	2015
0.893	1.15	5GOD:A	1	2016	0.886	3.82	4ZUX:C	1	2015
0.893	1.15	5GOB:A	1	2016	0.886	1.96	3HM3:D	2	2009
0.893	2.4	5EYA:C	1	2015	0.885	1.96	3HM3:B	2	2009
0.893	2.05	4PQT:B	1	2014	0.885	1.85	3ALB:B	1	2010
0.893	1.15	5GOD:B	1	2016	0.884	2.34	5FER:F	1	2015
0.893	2.4	5EYA:D	1	2015	0.884	2.21	4AP4:F	2	2012
0.892	1.85	3ALB:C	1	2010	0.883	2.4	3VHT:C	1	2011
0.892	1.26	3NHE:B	2	2010	0.883	1.55	5J8P:A	1	2016
0.892	3.82	4ZUX:M	1	2015	0.883	2.21	5AIU:F	1	2015
0.892	1.4	3A9K:B	2	2009	0.883	2.2	2XEW:I	2	2010
0.892	3.82	4ZUX:H	1	2015	0.883	1.9	3H7P:B	1	2009
0.891	1.7	3VUX:B	1	2012	0.882	1.75	1WRD:B	1	2004
0.891	3.48	5EDV:G	1	2015	0.882	2.21	4AP4:C	1	2012
0.891	2.1	5DFL:B	1	2015	0.881	2.35	3UGB:B	1	2011
0.891	1.85	2HD5:B	1	2006	0.881	2.39	3AUL:A	1	2011
0.891	1.9	3WWQ:K	1	2014	0.880	1.96	3HM3:C	2	2009
0.890	1.9	3WWQ:E	1	2014	0.880	2.7	4BVU:C	1	2013
0.890	1.95	2JF5:B	2	2007	0.880	2.34	5FER:C	1	2015
0.890	1.85	2QHO:C	2	2007	0.880	1.59	5GOI:A	2	2016

Table S1. Top Scoring Crystal Structures

This list includes all of the structures used to generate the 22-member high resolution crystal structure ensemble (≤ 1.74 Å resolution, R_u = 0.915).
